# Perceptions, Beliefs, and Knowledge of Oral and Familial Cancer in an Indigenous Community of Chile: A Mixed Quantitative—Qualitative Study

**DOI:** 10.1177/24731242251372703

**Published:** 2025-08-29

**Authors:** Cynthia Cantarutti, Gerardo Yévenes, Águeda Muñoz-del-Carpio-Toia, Daniela Adorno-Farias, Ricardo Fernández-Ramires, Alan Roger Santos-Silva, Jean Nunes dos Santos, Ignacio Molina-Ávila, Francisco J. Bravo, Wilfredo Alejandro González-Arriagada

**Affiliations:** ^1^ Escuela de Odontología, Pontificia Universidad Católica de Chile, Santiago, Chile.; ^2^ Escuela de Odontología, Universidad Arturo Prat, Iquique, Chile.; ^3^ Vicerrectoría de Investigación, Universidad Católica de Santa María de Arequipa, Arequipa, Perú;.; ^4^ Facultad de Odontología, Universidad de Chile, Santiago, Chile.; ^5^ Facultad de Ciencias, Universidad Mayor, Santiago, Chile.; ^6^ Oral Pathology, Oral Diagnosis Department, Piracicaba Dental School, State University of Campinas, Campinas, Brazil.; ^7^ Faculdade de Odontologia, Universidade Federal de Bahía, Salvador-Bahía, Brazil.; ^8^ Stomatology, Department of Dentistry, Hospital Dr. Arturo Oñativia, Salta, Argentina.; ^9^ Facultad de Odontología, Universidad de los Andes, Las Condes, Chile.; ^10^ Centro de Investigación e Innovación Biomédica, Universidad de los Andes, Las Condes, Chile.

**Keywords:** oral health, indigenous population, oral cancer, knowledge, cancer, health disparities, public health, racial minority, perception, beliefs

## Abstract

**Background:**

Oral cancer is one of the 10 most common cancers globally and represents a public health problem. Cultural practices and access to care are recognized as determinants of oral diseases, including cancer. Understanding the perceptions of indigenous communities is crucial for developing culturally appropriate interventions. This study aims to evaluate the perceptions, beliefs, and knowledge about oral and familial cancer within a specific indigenous community (Quechua and Aymara) in Chile using a mixed-methods approach.

**Methods:**

This exploratory study was conducted in two phases: an oral cavity clinical examination and a qualitative phase, consisting of semi-structured interviews with a subset of participants from the clinical examination. A total of 77 volunteers with no prior history of oral cancer underwent an oral cavity clinical examination, and 53% reported a familial history of cancer. The interview was conducted with 18 participants.

**Results:**

No significant differences were found in the clinical oral health status between the indigenous and nonindigenous populations. However, a lack of knowledge of oral cancer was noted in the indigenous community. A higher proportion of participants from the indigenous population reported a family history of cancer.

**Conclusion:**

The community demonstrated limited knowledge about oral cancer. Therefore, it is necessary to implement culturally and linguistically appropriate strategies for oral health promotion and oral cancer prevention to address the specific needs of these communities.

## Introduction 

Oral cancer is one of the 10 most common cancers worldwide and is a significant public health concern due to its high morbidity and mortality. The prevalence varies significantly worldwide, among different countries, age groups, sexes, races, and ethnicities.^1^ According to GLOBOCAN estimates for 2020, there were over 377,000 new cases of oral cancer, accounting for ∼2.1% of all new cancer cases, and about 177,000 deaths worldwide were associated with this disease.^2^ These global statistics underscore the need for targeted cancer prevention strategies. Indigenous populations face significant oral health challenges, including a notably high prevalence of pre-cancerous lesions, reported at 48.3%. This population is also at an increased risk for oral cancer, which is exacerbated by factors such as higher rates of smokeless tobacco and other carcinogenic leaf use.^3^ A study with indigenous population of Queensland reported a slightly lower prevalence of oral cancer (3.1%) compared with the overall population (4.6%). Despite this, indigenous populations experience significantly worse survival rates and prognoses compared with their nonindigenous counterparts, underscoring a critical disparity in health outcomes that necessitates targeted public health interventions.^4^

Ethnicity is a sociocultural construct based on shared cultural and historical traits.^5^ Ethnicity captures social, cultural, and environmental factors influencing health outcomes.^6^ Belonging to an indigenous community has been associated with poor health conditions, showing significant differences compared with the nonindigenous population. These communities often face considerable challenges in accessing health services because of their remote locations and lack of adequate infrastructure, creating barriers to both general and dental care.^1,7^ Although the cultural practices and lifestyles of indigenous communities can act as protective mechanisms against poor health, highlighting the importance of healthy habits and traditional practices for maintaining well-being, there are numerous factors that adversely influence their health.^1,7,8^ Furthermore, historical policies and practices have led to continuous marginalization and disadvantages for these populations. This lack of autonomy and low self-esteem resulting from these policies are directly associated with poorer health outcomes.^7^

Oral and pharyngeal cancer in Chile remains a significant health concern, accounting for 1% of all cancer deaths in the country between 1995 and 2002, with mortality rates increasing from 0.9 to 1.3 per 100,000 inhabitants during this period. The male-to-female ratio of deaths was 2.8:1, with the highest mortality observed in men aged 55–64 years and women over 75.^9,10^ A more recent study from 2002 to 2010 reported a stable mortality rate for oral and pharyngeal cancer, ranging from 1.11 to 1.25 per 100,000 inhabitants, with a male-to-female ratio of 2.3:1. The most frequent anatomical locations for these cancers were the tongue (17.7% of deaths), salivary glands, and oropharynx.^11^ From 2002 to 2012, 1611 deaths due to oral cancer were recorded, with a mean age of 67.6 years, and 63% of the deaths occurred in men. The adjusted mortality rate was 0.85 per 100,000 inhabitants, which was higher in men (1.13) than in women (0.58).^9^

In Chile, the indigenous population represents 12.8% of the total population according to the latest Population and Housing Census of 2017.^12^ The Aymara are a significant indigenous group primarily inhabiting the northern regions of Arica and Parinacota, Tarapacá, and Antofagasta, constituting 7.2% of the country’s indigenous population and being the second largest group after the Mapuche, who are concentrated in southern Chile. Quechua is another indigenous ethnic group present in northern Chile.^13^ At the local level, indigenous communities in Chile have a significant prevalence of oral health problems, including periodontal diseases and dental caries, which often go untreated due to barriers in access and lack of resources.^14^ Furthermore, the limited awareness and knowledge about oral cancer in these communities exacerbate the situation, resulting in late diagnoses and poorer prognoses.^15,16^

Health inequality between indigenous and nonindigenous populations reflects persistent structural and socioeconomic historical disparities.^17^ The need for culturally adapted strategies for cancer prevention and control in indigenous populations has been emphasized, highlighting the importance of actively involving these communities in the design and implementation of these strategies to ensure their effectiveness and cultural relevance.^18^

However, there is a significant research gap concerning how indigenous communities in Chile perceive oral and hereditary cancer. Despite the well-documented health disparities, little is known about how indigenous communities in Chile perceive oral and hereditary cancer, creating a critical gap in the design of effective public health interventions. Understanding the perceptions and beliefs of these communities is crucial for developing culturally relevant and effective prevention and treatment strategies for addressing health disparities and improving outcomes.

This research aims to determine whether the health inequities observed in indigenous populations are present in northern Chile, compared with urban populations. Specifically, this study sought to evaluate the perceptions, beliefs, and knowledge of oral and familial cancer in an indigenous community via a mixed-methods approach.

## Methodology

### Study design, ethical approval, and participants

A cross-sectional, mixed quantitative–qualitative study was conducted in Pica, Región of Tarapacá, Chile, and received ethical approval from the Research Ethics Committee of Universidad de los Andes, Chile (CEC2023107) on March 5, 2024. The study population included adults who provided informed consent to participate. The quantitative phase involved oral cavity clinical examinations performed by calibrated examiners. A subset of these individuals was subsequently selected for the qualitative phase, which consisted of semi-structured interviews. Inclusion criteria were adult (older than 18 years old), residency in Pica, and Spanish language proficiency. Exclusion criteria were adults with communication difficulties (assessed through observation during initial contact and the consent process) and dental stress or anxiety (evaluated via a brief, informal inquiry before the examination).

### Sample size

A total of 77 volunteers underwent an oral cavity clinical examination. For the interviews, convenience sampling was applied, aiming for data saturation with a subsample of 10 participants in the operation, seeking data saturation, but, finally, 18 individuals were included in the qualitative study.

### Data collection

#### Clinical examination

The oral cavity clinical examinations were conducted by sixth-grade dental students from Universidad Arturo Prat, Chile, who were calibrated throughout their professional training, with their clinical professors serving as the gold standard for ensuring adequate calibration. While inter-examiner reliability was not formally obtained, the ongoing clinical evaluation during their studies served as a continuous measure of their competence.

These examinations took place during dental procedures at one community center, utilizing mobile dental chairs equipped with sterile materials (dental mirror, periodontal probe, gauze, tongue depressor) and adequate illumination provided by dental examination lamps on the mobile units. The examination protocol involved a systematic visual inspection of the lips and extraoral tissues, followed by an evaluation of the buccal mucosa (right and left), gingiva (upper and lower, buccal and lingual/palatal), tongue (dorsal, ventral, lateral borders), floor of the mouth, and hard and soft palate, with all findings meticulously recorded in a dental record.

The student examiners were under the constant supervision of oral medicine specialists who provided immediate assistance for any questions or difficulties encountered during the examinations. Volunteers requiring a biopsy were referred to the dental service of the university. In addition to the clinical examination, complementary clinical data were collected from patients’ charts, and a survey based on questions from the ENS (National Health Survey, 2017) and ENCAVI (Chilean Quality of Life and Health Survey, 2015–2016), including the OHIP-7Sp, was also administered, to get data about their current employment status, highest educational level, country of birth, belonging to or descent from an indigenous group, smoking habits, perception of their oral health, history of dental visits and reasons, presence of oral discomfort, self-rated health and overall quality of life, and family history of cancer.

#### Semi-structured interviews

The qualitative analysis was guided by an interpretivist paradigm, using semi-structured interviews conducted to explore participants’ experiences, beliefs, knowledge, and perspectives about oral health and cancer within their cultural context.

The interview guide (Supplementary Data S1) was developed collaboratively by researchers with expertise in dental public health and qualitative methods. The development of this guide involved input from the research team, comprising experts in dental public health and qualitative research methodologies. Interviews were conducted in Spanish, as this is the primary language spoken by the participants in the study, and audio-recorded for transcription and analysis. Data were analyzed using inductive thematic analysis^19^ to identify, organize, and interpret patterns of meaning within the interview transcripts. Open coding was applied without predefined categories, and ATLAS.ti 24.1.1 software was used to assist data organization. The cultural context was considered during interpretation to better understand participants’ meanings.^20^ Triangulation by integrating qualitative interview findings with quantitative data from clinical examinations and surveys enhanced the credibility. Regular team discussions supported reflexivity, assessing saturation to ensure sufficient data, and addressing critical and nuanced data interpretation, mitigating potential researcher bias.

#### Operational definition of variables

The operational definitions of variables were as follows:

1. Rurality: Defined based on the participant’s primary place of residence as reported during the survey, according to the Chilean National Institute of Statistics (INE), as “rural” or “urban.”
2. Educational level: Defined as the highest level of formal education completed by the participant, categorized as follows: “prescholar,” “elementary,” “secondary,” “technical,” and “professional.”
3. Oral mucosal lesions: Defined as specific, clinically observable abnormalities of the oral mucosa identified during the clinical examination by calibrated examiners. These included oral cancer, oral leukoplakia, oral lichen planus, infectious disease (viral, fungal, or bacterial), and other.
4. Familial history of cancer: Defined as having at least one first-degree relative (parent, sibling, or child) who had been diagnosed with any type of cancer at any point in their lives, as reported by the participant during the survey (see Supplementary Data S2). Participants were asked a direct question, and a “yes” or “no” response was recorded.
5. Knowledge about cancer: Assessed using nine-item questionnaire included in the survey (see Supplementary Data S1). This questionnaire evaluated the participant’s understanding of basic concepts related to cancer, such as its definition, common risk factors (e.g., smoking), and general symptoms.
6. Knowledge about human papillomavirus (HPV): Assessed during the survey as spontaneous mention of the volunteers.

### Statistical analysis

The data were tabulated and analyzed using SPSSv.25, and descriptive statistics were used. Differences with a *p* value <0.05 were considered statistically significant. Our statistical analysis was primarily descriptive, focusing on summarizing sample characteristics (frequencies, percentages, means, standard deviations) to describe perceptions and other variables. Given the exploratory nature of the study and our primary aim of describing perceptions in this specific population, formal hypothesis testing was not the focus.

## Results

### Clinical data of the participants

Seventy-seven participants were included in the clinical exam, and 18 of them were referred for qualitative analysis through semi-structured individual interviews, including 13 women and 5 men, ranging in age from 62 to 83 years. Table 1 provides an overview of the data from the clinical examination of oral mucosal lesions of the individuals included in the study. All interview participants resided in rural areas, and nine of them self-identified as members of an indigenous community (Table 2). The educational background varied; Quechua participants had educational levels ranging from elementary to professional education, while Aymara participants predominantly had elementary education. The semi-structured interviews were transcribed and analyzed, with an average length of 10 min and an average of 5 pages of transcripts.

**Table 1.** Analysis and Distribution of Clinical and Sociodemographic Characteristics According to Ethnicity

Parameter	Ethnicity			*p* Value^b^	Total
	Aymara *n* (%)	Quechua *n* (%)	No/other^a^ *n* (%)		
Gender					
Male	6 (30)	6 (35.3)	16 (40)		28
Female	14 (70)	11 (64.7)	14 (70)	0.74	49
Age					
<70 years old	10 (50)	10 (58,8)	13 (32.5)		33
≥70 years old	10 (50)	7 (41.2)	27 (67.5)	0.14	44
Rurality^c^					
Urban	2 (10)	1 (6.3)	3 (7.7)		6
Rural	18 (90)	15 (93.8)	36 (92.3)	0.91	69
Educational level					
Prescholar	1 (5)	0 (0)	0 (0)		1
Elementary	9 (45)	3 (17.6)	15 (38.5)		27
Secondary	5 (25)	6 (35.3)	12 (30.8)		23
Technical	4 (20)	6 (35.3)	8 (20.5)		18
Professional	1 (5)	2 (11.8)	4 (10.3)	0.53	7
Oral mucosal lesions					
No	14 (70)	14 (82.4)	29 (72.5)		57
Cancer	0 (0)	0 (0)	0 (0)		0
Leukoplakia	0 (0)	0 (0)	1 (2.5)		1
Lichen planus	0 (0)	0 (0)	0 (0)		0
Infectious disease	1 (5)	1 (5.9)	2 (5)		4
Other	4 (20)	1 (5.9)	7 (17.5)		12
Nonregistered	1 (5)	1 (5.9)	1 (2.5)	0.936	3
Familial cancer					
Yes	9 (45)	12 (70.6)	20 (50)		41
No	10 (50)	5 (29.4)	17 (42.5)		32
Does not know	1 (5)	0 (0)	3 (7.5)	0.471	4
Total	20	17	40		77

^a^ No = participants who did not identify as belonging to any indigenous ethnic group; Other = participants who self-identified with indigenous groups other than Aymara or Quechua (1 Mapuche and 1 Diaguita).

^b^ Chi-square test.

^c^ Only 75 individuals gave the information (two participants were lost).

**Table 2.** Characterization of Participants in Qualitative Study

ID interview	Age	Gender	Ethnicity^a^	Educational level^b^
1	74	Female	No	Basic
2	81	Female	No	Basic
3	83	Male	Aymara	Media
4	72	Female	Quechua	Basic
5	80	Female	No	Basic
6	71	Male	No	Basic
7	75	Female	Quechua	Professional
8	74	Female	No	Media
9	63	Female	Diaguita	Media
10	66	Male	Quechua	Primary
11	62	Female	No	Media
12	79	Female	No	Basic
13	63	Female	Aymara	Technical
14	64	Female	Quechua	Technical
15	81	Female	No	Basic
16	66	Female	Quechua	Media
17	67	Female	No	Basic
18	67	Female	Aymara	Basic

^a^ No = participants who did not identify as belonging to any indigenous ethnic group.

^b^ Level of education was grouped into six levels according to those that appear in the Supplementary Data S2.

Basic, elementary school; Media, scientific-humanistic secondary education or commercial, industrial, or teaching secondary education or technical professional secondary education; No, never attended; Primary, nursery, infant school, prekindergarten/kindergarten; Professional, professional (careers of 4 or more years) and postgraduate; Technical, higher level technician (careers of 1–3 years).

The analysis revealed no significant differences in the prevalence of oral mucosa lesions between indigenous and nonindigenous populations (*p* = 0.936). In addition, a higher proportion of participants who self-identified as Quechuas had a history of familial cancer (*n* = 12 out of 17, 70.6%) compared with Aymara (*n* = 9 out of 10, 45%) participants (*p* = 0.471).

The gender distribution showed that the majority of participants were female (64.7% for Quechua and 70% for “other”), with no significant difference between groups (*p* = 0.74). The age distribution indicated that a greater proportion of participants aged 70 years or older belonged to the “other” group (67.5%) compared with the Quechua (41.2%) and Aymara (50%) groups (*p* = 0.14). The analysis of the responses regarding quality of life did not reveal any statistically significant differences between the study groups (indigenous and nonindigenous populations).

### Rurality and educational level

Most participants resided in rural areas, with 93.8% of the participants in Quechuas and 92.3% of the participants in the “other” group living in rural settings (*p* = 0.91). Regarding educational level, 35.3% of the Quechua participants had secondary education, and 35.8% of the participants in the “other” group had elementary education (*p* = 0.53).

### Oral mucosal lesions and familial cancer

No participants were diagnosed with oral cancer. One participant from the “other” group presented with leukoplakia, and 5.9% of Quechua and 5% of Aymara participants presented with infectious diseases. Familial cancer was reported by 70.6% of the Quechuas and 45% of the Aymaras (*p* = 0.471).

### Perceptions, beliefs, and knowledge about oral cancer

The participants reported knowing people who have suffered from cancer. They know about the existence of breast cancer and cancer at other sites, such as the uterus, prostate, lung, stomach, and colon. However, most do not know that oral cancer exists and do not know anyone who has suffered oral cancer.

The participants associated neglect or lack of oral hygiene with a greater risk of having oral cancer. They also believed that when they have wounds that are not cared for and consequently do not heal, they could have a greater risk of developing cancer. Additionally, they considered that not going to the dentist was also a risk factor, but they stopped going to the dentist because they started using dentures.

When asked how they imagined oral cancer looks, they describe that it must be horrible, that they cannot imagine it, or that it may be black gums, teeth that start to fall out, a painful and deep wound, strange and red bumps, or disfiguration of the face. On the contrary, some people indicate that they believe it is something that is not seen because it is inside the mouth and that area is not visible. They state that they do not truly know what oral cancer looks like, but they assume that oral cancer would cause difficulties in eating. One participant mentioned that she has seen what oral cancer looks like on social media and that practicing oral sex is a risk factor, but she does not associate it with any type of virus.

*I think it must look horrible, not something that would not even let you drink water. The pain would be excruciating because, as I said, because when you see a person who has cancer and see the pain and suddenly the bad smells, because even if they are being treated, bad smells come out. I imagine that the mouth must be the same*. (E9, female, 63 years old, Diaguita)

*I believe that due to poor care of some, or rather a tooth or molar that is not cleaned well or that cavities are not filled, or rather poor care*. (E16, female, 66 years old, Quechua)

### Perceptions, beliefs, and knowledge about the HPV

Most people do not relate the papillomaviruses to oral cancer but rather to cancer in general. They had gained this information because of vaccination campaigns where their relatives, mostly grandchildren, had received the vaccine against the virus.

*Papilloma, that is papilloma (in response to the question, have you heard that any virus causes cancer). My grandson was given a vaccine against papilloma.* (E11, woman, 62 years old, nonindigenous)

They do not know about oral cancer, but they do know that because of cancer, there are manifestations in the mouth, this from their own experience or from family members or close people who have lived with the experience of suffering from cancer.

*No, I do not know it (oral cancer), but I have seen people with… you can see they have problems in the… in the mouth due to cancer*. (E10, man, 66 years old, Quechua)

*So now with the issue of my cancers, the oncologist has, he is the one who has to give the go-ahead for the operation on me, to remove my tooth, because yesterday he was explaining to me, the doctor was saying that if they had given me a medication that right now I do not, I do not know what it is called, they could not remove my tooth, why? Because there’s a hole and that hole does not close.* (E13, woman, 63 years old, Aymara)

### Perceptions, beliefs, and knowledge about how to take care of oral health

They reported that for the care of wounds in the mouth, they use mouthwashes or topical application of alcohol, hydrogen peroxide, vinegar, baking soda, plantain, or lemon, maintain dental hygiene, and wait for it to go away.

In the face of an ulcer of longer duration, such as 2 or 3 weeks, they report that they would visit the dentist or doctor seeking help.

## Discussion

This mixed-methods study, conducted in an indigenous community in northern Chile, revealed no significant differences in clinical oral health status between indigenous and nonindigenous participants. However, a notable lack of knowledge regarding oral cancer was observed within the indigenous community, alongside a higher proportion of self-reported family history of cancer in this population. Our study found a reported family history of cancer of 70.6% for the Quechua and 45% for the Aymara groups (Table 1), compared with the prevalence of 20–25% of family history of breast cancer in the general Chilean population in the literature,^21,22^ and the 50% self-reported in our study by the non-Quechua and non-Aymara population.

Membership in an indigenous community has been reported as a determinant of poor oral health. These reports have highlighted factors such as historical inequities, difficulties in accessing dental care, rurality, educational level, and cultural beliefs.^17,23,24^ Our study did not find differences in oral mucosal lesion examinations between indigenous communities and nonindigenous population. Importantly, over the past decade, the Quechua community has been in a process of ethnic recognition or de-aymarization^25^ because Chile previously identified all indigenous communities in the north as Aymara, leading Quechua descendants to reclaim their culture and language.^26^ This process could lead to the identification of differences related to ethnicity, such as the greater likelihood of familial cancer in the Quechua community than that reported by Aymaras.

Our results revealed no differences in the prevalence of oral mucosal lesions between indigenous and nonindigenous populations, which may be due to the relatively small sample size or relatively recent improvements in access to oral health services in rural communities. The sample size and the lack of a formal power analysis of the sample are recognized as limitations of our study. Although historical inequities in health care access continue to affect these populations, it would be valuable to investigate whether improvements in primary health care services in rural areas have contributed to more equitable diagnoses of oral conditions. It is important to note that a high percentage of participants resided in rural areas (92.3%), and a significant proportion had low educational levels (69.3%, used as a proxy for poverty) (Table 1), factors that could potentially confound the attribution of our findings primarily to ethnicity. However, we did observe differences in familial cancer history between the indigenous groups (Quechua 70.6%, Aymara 45%) and the “other” group (50%), suggesting a potential influence of ethnicity independent of rurality. We suggest that future research with a more balanced representation of urban and rural participants across different socioeconomic strata is needed to further explore these interactions and attribute findings to ethnicity. Another limitation of our study was the absence of numeric data on participants’ attendance at dental checkups in the past year, particularly in the context of our discussion regarding access to primary health care. Our survey (Supplementary Data S2) did include questions that provide some insight into this area. Specifically, we asked participants: “When was the last time you visited the dentist? (Not including today’s visit),” “Main reason for your last visit to the dentist,” and “In the last 6 months, did you receive dental care? (Without considering today’s visit).” However, our analysis of this survey data did not reveal any statistically significant differences in dental checkup attendance between the indigenous and nonindigenous populations in our sample.

The qualitative study revealed a lack of knowledge and an altered perception about oral cancer and how cancer looks. The participants of this study, despite knowing different types of cancer and/or knowing someone close to them who has suffered from a type of cancer, did not know that oral cancer existed. The patients were familiar with breast, uterus, prostate, lungs, and stomach. This lack of knowledge is consistent with previous studies in which the population was unaware of the disease.^27,28^ Additionally, this group reported that this pathology could appear in the mouth in different ways, such as black gums, tooth loss, or sometimes is undetectable; this finding agrees with previous studies that reported the inadequate knowledge regarding how the disease presents itself.^29,30^ Given this, it is possible that people are not able to detect warning signs if they self-examine, as found in the literature, where it is reported that they would not know what to look for when performing a self-examination.^31,32^ Finally, with respect to knowledge about risk factors, the people reported that they believe that a lack of hygiene or wounds that did not heal increased the risk of suffering from oral cancer. The literature reports that a low proportion of people associate alcohol consumption with the risk of suffering from oral cancer.^33^ Therefore, in line with the suggestions of the available evidence, it is necessary that people be trained about this disease in terms of its risk factors and warning signs to carry out adequate prevention.

The low level of awareness of oral cancer observed in this study emphasizes the need for culturally adapted health education interventions. Such interventions should be tailored to indigenous and rural communities, with a focus on improving knowledge about oral cancer symptoms, risk factors, and prevention. Increasing awareness through culturally relevant campaigns could help promote earlier detection and reduce the morbidity associated with late diagnoses.

A strength of this study is its incorporation of a qualitative methodology in indigenous communities in oral cancer. Understanding the knowledge, attitudes, and practices in these communities allows the development of strategies to improve oral health.^34^ A limitation of the current study could be selection bias for both the clinical examination and the qualitative phase because although participants share many characteristics, they are patients seeking care, which overestimates their knowledge in comparison with those patients who do not look for oral health. People with greater knowledge about oral health are more likely to visit a dentist.^35,36^ In Chile and many countries, indigenous identity is based on self-identification,^37^ which could be another bias in these reports. Additionally, the communities in northern Chile are generally well-integrated into the general population,^38^ unlike the isolated tribal communities in Amazonia or Oceania.^24^

In conclusion, our study did not evidence statistically significant differences in clinical oral health status between indigenous and nonindigenous participants, suggesting that the lack of knowledge about oral cancer is generalized to older adults in this community. Differences were noted in the proportion of familial cancer, which was greater in Quechua participants than in Aymara participants and the general population. It is important to consider the inclusion of cultural aspects for oral cancer education and prevention strategies tailored to the specific needs of indigenous communities.

## Abbreviations Used

ENCAVI: Encuesta Nacional de Calidad de Vida (Chilean Quality of Life and Health Survey)
ENS: Encuesta Nacional de Salud (National Health Survey)
HPV: Human papillomavirus
INE: Instituto Nacional de Estadística (National Institute of Statistics)
OHIP: Oral health impact profile
