# Supplementary material for: Perceptions, Beliefs, and Knowledge of Oral and Familial Cancer in an Indigenous Community of Chile: A Mixed Quantitative—Qualitative Study
Source: Health Equity. 2025 Aug 29;9(1):433–41. doi: 10.1177/24731242251372703 (PMC12412389; doi:10.1177/24731242251372703)
Supplement: Supplementary Data S1 [file 24731242251372703_supp_datas1.pdf]

Interview Instrument

INTERVIEW SCRIPT

Instructions to the interviewer: The text highlighted in yellow indicates instructions to better guide the interview.

Read the following text for the informed consent process.

Before starting, make sure you are recording the session.

Hello, Good morning/afternoon. How are you?

Read the informed consent of the interview.

Thank you very much.

This interview will be recorded and transcribed. Would you have any problem with that?

Do you have any questions regarding the informed consent that was previously developed?

It would be of great interest for us to know your perceptions regarding the aforementioned topic, for which we will ask some questions:

In terms of organization, we will begin by asking you some general questions about our topic of interest.

1. What does having good or bad oral health mean to you? Who determines if someone has a healthy mouth?
2. Do you consider that you have good or bad oral health? Why?
3. What aspects of life are affected when a person does not have good oral health? What aspects are favored when there is good oral health? And in your case, have you experienced those aspects being affected or favored?
4. When we talk about diseases of the mouth, what diseases do you know? Or, what parts of the mouth get sick?

5. When do you think it is necessary to see a dentist?
6. How often do you go to the dentist? When you go to the dentist, do you do all the recommended treatments or only treat the most urgent problem?
7. Do you know what cancer is?
8. What types of cancer do you know?
9. Have you heard of oral cancer?
10. Do you know anyone who has had cancer in their mouth? What do you know about what that illness was like? Do you think oral cancer is common?
11. Are you aware of anything that increases the risk of getting oral cancer? What?
12. Have you heard that any virus is related to getting cancer? And related to oral cancer?
13. What do you think oral cancer looks like?
14. When you have a wound in your mouth, what do you do?
15. If you had an ulcer in your mouth for more than three weeks, what would you do?

Now I will ask you some questions regarding your experience when you were a child or teenager:

1. Based on your experience, what was oral care like in the past? What were you accustomed to doing in your family when you were a child to take care of oral health and to deal with diseases of the mouth? (The idea is to ask further questions about how the care practices were carried out and who was in charge.)
2. In your perception, has this changed? Since when? Has it been for better or for worse? What do you think this change is due to? I am referring to the way in which problems of the mouth and teeth are cared for and solved.
3. Would you like to comment on anything else regarding the topic we have been discussing?
4. Do you have any children under the age of 9 in your care? If yes, continue with the questions in the Caregiver Survey section. If no, end the interview with the following statement:

Thank you very much for your time. Just remember that the results of the research you are collaborating with will be of great help in making decisions when implementing oral health programs in health care centers, such as the center where you are currently seen.

Caregiver Survey

Now I will ask you some questions about your current experience as a caregiver of a child:

1. Why do you think oral diseases occur?
2. What actors/people/sources of information/factors in general have influenced the habits you have in your home and with the child in your care? (If they don't understand what we mean by "actors," we can say, where have you obtained information about your own and the child's oral health care?)
3. Do you recognize that there are elements that generate a greater risk or that cause oral diseases in children? What are they?
4. Do you consider that you know how to maintain the oral health of the child in your care? What strategies do you use at home?
5. How responsible do you feel for the oral health of the child in your care? Are there other people/entities responsible?
6. If it were 100% up to you, would the child in your care have a healthy mouth? Do you feel prepared and capable of maintaining the child's mouth healthy? (Do they consider that if it is in their hands they can keep their children healthy, how can they do it? How far/close are they to achieving it?)
7. How does your parenting style relate to the general and oral health care of the child in your care? (Investigate if there are routines, house rules.) What are the family's behaviors regarding habits? Does everyone take care of themselves, or is this isolated and only focused on the child?
8. How does the daycare/school support the general and oral health care of the child in your care? Do they teach the children? What do they teach them?

Now I will ask you some questions regarding your experience as a person who is responsible for the care of a child who is seen at the dentist in a Cesfam or clinic (clarify that the experience is as a caregiver of the child who is a user).

1. Is the child in your care under control at the dentist in the clinic or Cesfam? When was their last appointment and when are they due to return?
2. Have you had difficulties taking them to dentist appointments, or getting a check-up appointment? What difficulties have you had?
3. When the dentist who treats the child in your care finishes a session, do they give you care instructions? Do you remember any instructions they have given you?
4. Have these instructions seemed possible to follow? Is there any instruction that you consider impossible to comply with or that you do not want to follow? Why?
5. How was the experience with the dentist? Did they involve you in decision-making or did they only give you instructions? Did they listen to your points of view?
6. Do you consider that you will follow the instruction to attend the check-up on the scheduled date? Why?
7. Do you consider that your previous experiences as a patient at the dentist (whether good or bad) influence the experience of the child in your care? How do they influence it?
8. If I tell you that today the focus of dental care is centered on the person and that for this it is necessary for patients or users to have a leading role, taking responsibility for their health and the health of those in their care, what do you think about this? What impressions or apprehensions does it generate for you?
9. Can you identify yourself as a person committed to your oral health and that of the child in your care? What characteristics indicate that you are a committed patient, or what do you lack to feel that way?
10. If the goal is to keep the child in your care with a healthy mouth, how do you monitor that this is achieved?

Thank you very much for your time. Just remember that the results of the research you are collaborating with will be of great help in making decisions when implementing oral health programs in health care centers, such as the center where you are currently seen.
