# Supplementary material for: Perceptions, Beliefs, and Knowledge of Oral and Familial Cancer in an Indigenous Community of Chile: A Mixed Quantitative—Qualitative Study
Source: Health Equity. 2025 Aug 29;9(1):433–41. doi: 10.1177/24731242251372703 (PMC12412389; doi:10.1177/24731242251372703)
Supplement: Supplementary Data S2 [file 24731242251372703_supp_datas2.pdf]

**Questionnaire**

ID:

Interviewee's First Name: _____ Middle Name: _____ Last Name: _____

Date of Birth: _____ Age in Years: _____

Address: Rural ☐ Urban ☐

Sex: Male ☐ Female ☐

---

What is your current employment status?

Employed (with a boss or employer) ☐

Self-employed (entrepreneur or own account) ☐

Unemployed ☐ First-time job seeker ☐

Retired ☐ Homemaker ☐

Inactive ☐ Don't know / No response ☐

---

What is your highest level of education attained, or current level? (ENS, 2017)

Never attended | Nursery | Infant School | Prekinder/kinder | Special Education |

Elementary School | Scientific-humanistic secondary education |

Commercial, industrial, or teaching technical education (old system) |

Technical professional secondary education |

Higher level technician (careers of 1 to 3 years) |

Professional (careers of 4 or more years) |

Postgraduate | Don't know | No response

---

What country were you born in? (ENS, 2017)

Chile | Other |

If other country, what country? | How long have you lived in Chile? |

Years/Months | How long have you lived in this region? | Years/Months |

---

In Chile, the law recognizes nine indigenous peoples. Do you belong to or are you descended from any of them? (ENS, 2017)

Aymara ☐ Rapa Nui ☐ Quechua ☐ Mapuche ☐ Atacameño (Likan Antai) ☐

Coya ☐ Kawésqar (Alacalufes) ☐ Yagán (Yámana) ☐ Diaguita ☐

Does not belong to any Chilean indigenous people ☐

---

**Smoking**

Do you currently smoke cigarettes? (ENS, 2017)

Yes, one or more cigarettes a day ☐

On average, how many cigarettes did you smoke per day during the last 30 days?

Number of cigarettes per day

At what age did you start smoking?

Don't remember/not sure

Yes, occasionally (less than one cigarette a day) ☐

No, I have quit smoking ☐

No, I have never smoked ☐

---

**Oral Health**

In general, how would you rate your oral health? (ENS, 2017)

Very good ☐ Good ☐ Fair ☐ Poor ☐ Very poor ☐

---

When was the last time you visited the dentist? (Not including today's visit)

Less than 6 months ago ☐

Between 6 months and a year ☐

More than 1 year and less than 2 years ☐

2 or more years but less than 5 years ☐

5 or more years ☐

---

Main reason for your last visit to the dentist (ENS, 2017)

Pain or discomfort in teeth, gums, or mouth ☐

Treatment / Continue treatment ☐

Check-up or routine control ☐

Don't know ☐

No response ☐

---

In the last 6 months, did you receive dental care? (Without consider today's visit)

Yes ☐

No, requested care, was given an appointment, but could not attend ☐

No, requested care and was not given an appointment ☐

No, needed care but did not request it ☐

Did not need care ☐

---

Have you been in any of the following situations? (ENS 2017)

My teeth or dentures bother me when I speak

Never | Almost Never | Sometimes | Almost Always | Always |

My teeth or dentures cause suffering and pain

Never | Almost Never | Sometimes | Almost Always | Always |

My teeth or dentures make me uncomfortable when I eat

Never | Almost Never | Sometimes | Almost Always | Always |

My teeth or dentures interfere with my daily activities (work, study, housework, etc.)

Never | Almost Never | Sometimes | Almost Always | Always |

My teeth or dentures interfere with my social relationships

Never | Almost Never | Sometimes | Almost Always | Always |

---

**Quality of Life and Health (ENCAVI, 2015-2016)**

In general, would you say your health is:

Excellent | Very good | Good | Fair | Poor | Don't know | No response

How would you rate your quality of life?

Very poor | Poor | Neither good nor poor |

Good |Very good | Don't know | No response

---

**OHIP-7SP (ENCAVI, 2015-2016)**

Has your digestion worsened due to problems with your teeth, mouth, or dentures?

During the last year: Never | Almost Never | Sometimes | Almost Always | Always | Don't Know | No Response

Have you had sensitive teeth, for example, due to cold foods or liquids?

During the last year: Never | Almost Never | Sometimes | Almost Always | Always | Don't Know | No Response

Have dental problems made you feel completely unhappy?

During the last year: Never | Almost Never | Sometimes | Almost Always | Always | Don't Know | No Response

Have people misunderstood any of your words due to problems with your teeth, mouth, or dentures?

During the last year: Never | Almost Never | Sometimes | Almost Always | Always | Don't Know | No Response

Has your sleep been interrupted by problems with your teeth, mouth, or dentures?

During the last year: Never | Almost Never | Sometimes | Almost Always | Always | Don't Know | No Response

Have you had difficulty doing your usual work due to problems with your teeth, mouth, or dentures?

During the last year: Never | Almost Never | Sometimes | Almost Always | Always | Don't Know | No Response

Have you been totally unable to function due to problems with your teeth, mouth, or dentures?

During the last year: Never | Almost Never | Sometimes | Almost Always | Always | Don't Know | No Response

---

In your family, is there a member who has had cancer, including yourself?

Yes, one member

Yes, more than one member

No

Don't know

What is the relationship? (You can mark more than one)

Yourself

Mother

Sister/Brother

Daughter/Son

Maternal Grandparents

Maternal Aunts/Uncles

Paternal Grandparents

Paternal Aunts/Uncles
